# Smallholder farmers' willingness to pay for scale-appropriate farm mechanization: Evidence from the mid-hills of Nepal

**DOI:** 10.1016/j.techsoc.2019.101196

**Published:** 2019-11

**Authors:** Gokul P. Paudel, Dilli Bahadur KC, Dil Bahadur Rahut, Narayan P. Khanal, Scott E. Justice, Andrew J. McDonald

**Affiliations:** a International Maize and Wheat Improvement Center, South Asia Regional Office, Kathmandu, Nepal; b International Maize and Wheat Improvement Center, El Batan, Mexico; c Section of Soil and Crop Sciences, School of Integrative Plant Sciences, Cornell University, New York, USA

**Keywords:** Farm mechanization, Mini-tillers, Willingness to pay, Contingent valuation, Heterogeneous demand, Nepal

## Abstract

This paper analyzes smallholder farmers' willingness to pay (WTP) for the purchase of scale-appropriate farm mechanization in the hill ecologies of Nepal using the case of mini-tiller technology: a small, 5–7 horsepower two-wheel tractor primarily used for agricultural land preparation. Using primary survey data from 628 randomly-selected households, we find that farm size, local wage rates, out-migration, access to credit services, and associations with agricultural cooperatives positively influence the WTP for mini-tillers while the number of draft animals owned negatively influence the WTP for mini-tillers. On average, farmers were willing to pay 31% less than the actual price of a mini-tiller. Results also exhibited a heterogeneous demand in which the lowest quartile farm size households, typically the poorest farm households, were willing to pay 26% less for the mini-tiller than the top quartile of farms. In the context of labor scarcity and rising rural wages, agricultural policy on farm mechanization in Nepal should aim to prioritize small farms through robust service provision models in order to increase the level of farm mechanization in the country.

## Highlights

• Scale-appropriate mechanization has the potential to mitigate the problem of labor scarcity.
• Farm size, on-farm labor wages, and access to credit are positively associated with WTP for the mini-tiller.
• Number of draft animals owned is negatively associated with WTP for the mini-tiller.
• Farmers' average WTP was 31% lower than the actual mini-tiller price.
• Compared to the richest quartile farms, the WTP of the poorest quartile was 26% lower for mini-tillers.

## 1 Introduction

In developing countries, farm mechanization plays a significant role in augmenting the scale of farm operations, decreasing the cost of production, reducing drudgery, improving the timeliness of operations, enhancing crop productivity, and contributing to increases in household income [[1], [2], [3], [4]]. Farm mechanization has the potential to enhance crop productivity and improve food security and rural livelihoods in the developing world where small farms, low crop productivity, high food insecurity, and poverty are common [2,[5], [6], [7]]. Nevertheless, farm mechanization among smallholders is challenged by small and fragmented land holdings, lack of financial resources among smallholder farmers to invest in the technology, low risk-bearing capacity, and low levels of market integration [[8], [9], [10], [11], [12], [13]]. As smallholder farming systems are the dominant type of agriculture across South Asia and Sub-Saharan Africa, discussion has emerged on the types and models of farm mechanization that could improve farm efficiency, crop productivity, and food security.

Previous studies have revealed that agricultural development in smallholder farming systems across South Asia and Africa are challenged by labor-intensive farming, low labor productivity, low input uses, and low rates of return from farming [1,8,11,12,[14], [15], [16]]. Additionally, in recent years, the agriculture sector in many developing countries has been facing rapid labor out-migration [17], which has led to an acute labor shortage in the agriculture sector and delays in crop cultivation practices in countries like Nepal [[18], [19], [20]]. The labor shortage is further aggravated by rising rural wages [21]. Rising wages have led to an increase in the cost of agricultural production [22]. While the wage increases may be beneficial to the segment of the population that depends on the wages for income, the increase in the cost of production has eroded profit margins for many smallholders. In this context, policy planners in developing countries such as Nepal have recognized scale-appropriate mechanization as a core intervention that can help restore the economic viability of small farm enterprises.^1^ Many studies show that farm mechanization can play a crucial role in minimizing farm drudgery, decreasing the cost of production, and increasing profitability [1,3] and some studies have demonstrated an increased level of farm mechanization in the smallholder systems through service provision models [11,12,16,[23], [24], [25]]. However, the adoption of farm mechanization in hill production ecologies is challenged by rugged terrain as well as small and often terraced plots [26].

Mechanized tools are used for different types of crop cultivation and crop management practices. The most common operations include land preparation, mechanical seeding, threshing, and harvesting. Among these crop cultivation practices, land preparation (tillage operations) is the most important activity and is generally completed by human labor and/or animal traction in developing countries. Draft animals play an important role in agricultural land preparation, particularly tillage operations [27]. Nevertheless, among smallholder farmers in South Asia, there is a decreasing trend of keeping draft animals due to low livestock productivity, lack of sufficient feed, high costs of rearing livestock, lack of sufficient grazing and pasture land, decreasing farm size, and labor scarcity [28]. Consequently, many farmers are unable to plant crops on time. Fallows are also expanding due to the high cost of cultivation, scarcity of labor, rising labor wages, and associated drudgery related to farming [22]. Therefore, there is potential for scale-appropriate farm mechanization to overcome these problems and decrease the cost of production associated with farming while increasing farm profits and making agriculture more economically viable.

**Fig. F1:**
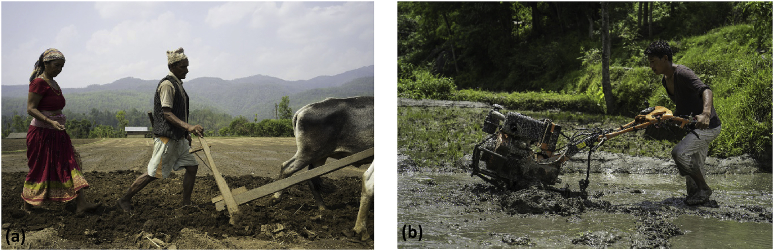
Methods of land preparation: (a) farmers using traditional method of land preparation through bullocks and indigenous plough and (b) farmers using mini-tillers for rice land preparation. Source: Peter Lowe/CIMMYT.

In this paper, we analyzed smallholder farmers' WTP for farm mechanization by assessing their willingness to purchase low-cost mini-tillers in the mid-hills of Nepal. A mini-tiller is a small, 5–7 horsepower tractor primarily used for agricultural land preparation, particularly tillage operations [29]. In Asia, mini-tillers are largely manufactured in China and India and are imported to Nepal [30]. The private sector in Nepal plays a key role in importing mini-tillers, mostly from China. The cost of the mini-tiller in the Nepalese market ranges from NPR 35,000 (US $337) to NPR 65,000 (US $625), depending on its horsepower. The sample images of the mini-tillers versus the traditional method land preparation can be found in Appendix (Fig. F1). Adoption of larger tractors is not broadly possible due to steep slopes, terraced plots, fragmented land, and lack of developed market infrastructure [31,32]. Mini-tillers can be easily transported from one plot to another, can operate in terraced plots, consume less fuel, and are easy for women to use due to their small size and light weight. To facilitate the scaling of this technology, our study examines farmers' WTP for the purchase of mini-tillers in the mid-hills of Nepal using the contingent valuation method.

## 2 Study background

Nepal is an agricultural country with almost two-thirds of its population depending on agriculture for their livelihoods [33]. The agriculture sector composes almost one-third of the national economy [34]. However, agricultural crop productivity in Nepal is the lowest among South Asian countries [35]. Food insecurity in Nepal is a major problem with more than two-thirds of 75 districts facing food deficits every year [36]. In recent years, the agriculture sector in Nepal has been facing an acute labor shortage due to rapidly increasing labor out-migration, especially migration to the Gulf countries by people in search of better employment opportunities. The labor shortage has increased the rural labor wage rates [21,25,37]. Increased labor out-migration, particularly by males, has also increased the responsibility of females and turned them into *de-facto* female-headed households [38,39].

Farm mechanization in Nepal formally started in the 1970s with the advent of two- and four-wheel tractors [31]. In the 1980s, Japanese two-wheel tractors entered Nepal, followed by the arrival of Korean and Chinese two-wheel tractors in the late 1980s [40]. Mechanization during the early period typically only referred to tractor tillage, until the arrival of threshers in the 1990s and combiners in the 2000s in Nepal's Terai region [8]. Before 2004, all mechanization-based interventions were concentrated in the plain areas and Terai region of Nepal [30]. Takeshima (2017) reported that less than 8% of farms used farm mechanization in the hills, while 46% of farms used mechanization in the Terai area.

In recent years, small-scale mechanization in Nepal has emerged as an important opportunity to increase agricultural productivity, especially after the promulgation of the farm mechanization policy in 2014 [31]. However, this policy was formulated without an in-depth understanding of scaling potential and program design principles that would facilitate broad adoption. In this context, we attempted to assess farmers' WTP for scale-appropriate farm mechanization to understand the demand for the technology in smallholder farming systems in the hill production ecologies of Nepal. We empirically estimated factors driving farmers' WTP for small-scale farm mechanization through a contingent valuation method. We used a semi-bound dichotomous choice model to gain insights on farmers' WTP for mini-tiller technology because it provides the real market situation to the farmers. We also derived differential demand curves for the mini-tillers across different farm size quartiles and socio-economic strata in the mid-hills of Nepal.

## 3 Materials and methods

### 3.1 Data

**Fig. 1:**
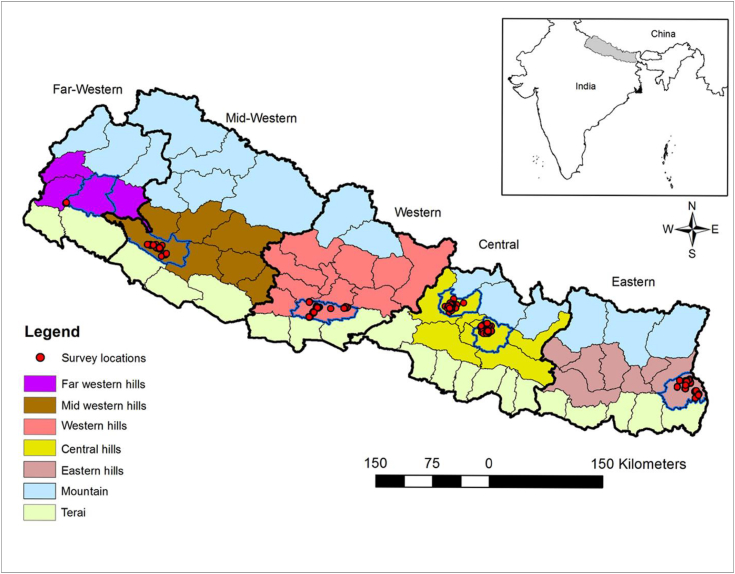
Map of Nepal showing the study districts and samples distribution.

The current study is based on primary household survey data collected from the mid-hills of Nepal from October to November 2017. The data was collected through face-to-face interviews with a structured questionnaire designed in an electronic software (www.surveybe.com; last assessed October 9, 2018) in order to minimize data entry errors and the time for the survey. The questionnaire included sections to elicit information on household socio-economic status, cropping systems, income sources as well as questions to elicit WTP for mini-tillers. The sampling strategy consists of a purposive selection of six districts across the mid-hills of Nepal. Districts were selected based on the potential for adoption of scale-appropriate farm mechanization. With very low levels of mechanization, these districts are homogeneous in terms of farming systems. The types of crop grown, livestock, remoteness, climate, and land typologies are similar across these districts. Maize is the primary crop grown in these districts, in addition to other crops such as rice, wheat, and vegetables. These districts were selected so that each would represent different developmental regions in the mid-hills.^2^ After consultation with district agriculture development officers and key informants in each district, a total of 29 sub-districts called Village Development Committees, or VDCs, were also selected purposively from the sampled six districts. In each of the VDCs, villages were selected randomly.^3^ Finally, a pool of 628 households were selected randomly for the survey from sampled VDCs and villages to elicit farmers' WTP for the mini-tillers. The distribution of the samples by districts and regions is presented in Fig. 1.

### 3.2 Analytical framework

Farmer perceptions of technology cannot be assessed without sound knowledge of the benefits of adoption [41]. WTP can be investigated either through revealed preferences or by stated preferences methods [42]. The revealed preferences method, however, is used when farmers have knowledge and insight about the technology and its associated benefits [43]. It may create a problem when farmers do not have sufficient information and incentives to thoroughly evaluate the values associated with such technology if a market were to exist, and therefore might not yield realistic estimates [44].

The stated preferences method, however, is easier for the farmers to understand and is more realistic since farmers are educated on expected benefits and can relate more to a real market situation [41]. With this method, two different econometrics approaches are generally used for assessing farmers' WTP for the technology: (i) single bound approach [45] and (ii) double bound approach [42]. In a single bound approach, farmers are able to provide the “yes” or “no” responses to the average market price of the mini-tiller technology. This approach is incentive-compatible since it is in the farmers' specific interest to say “yes” if their WTP is greater or equal to the market price of mini-tiller technology and to say “no” otherwise. However, Hanemann et al. [42] demonstrated that the single bound approach is statistically inefficient because of the large sample size requirement. The double-bound approach is generally preferred to increase the efficiency, since follow-up questions (higher and/or lower than initial price) are administered to the farmers.

In this study, while adopting a double bound approach, we first assessed the average market price for the mini-tiller based on the selling price of traders in a few urban areas as an initial price for the bidding. We only provided lower bids to the farmers who responded negatively to the initial price. This method is referred to as a one-and-one-half bound approach. Farmers who responded positively to the initial bid were not asked follow-up questions to establish an upper price point because prices of mini-tillers are expected to decline as markets develop. Similar approaches have been used in earlier studies to assess farmers' WTP for agricultural technologies [43,[46], [47], [48]]. Furthermore, Cooper et al. [49] demonstrated higher efficiency of the one-and-one-half bound approach than the double bound approach.

In order to derive the average market price for the bids, we researched the price of mini-tillers with different traders. The median price of the mini-tiller was NPR 55,000 (US $529). The bid structures to elicit the WTP for mini-tiller technology are shown in the Appendix (Table A1). Since the follow-up bids were offered only to the farmers who responded negatively to the initial bid, it is likely that farmers would say “no” to the second bids if the second bids were very close to the initial price. Therefore, we maintained a difference of 9% (NPR 5,000 or US $48) between the largest second bids and the initial price bid. However, a range of 3.6% (NPR 2,000 or US $19) was maintained for the rest of the biddings so that farmers could realize a level of discount on the mini-tiller.

A total of 12 possible follow-up bids were formulated, and the lowest bid was NPR 28,000 (US $269) which was equivalent to the mini-tiller manufacturer price. Also, this lowest bid resembles the average price for a pair of bullocks (agricultural land is conventionally prepared using bullocks), so this lowest contingent bid represents a technology price premium of zero. Finally, the second bids were randomly selected by the enumerators using electronic devices and offered to the farmers who responded negatively to the initial bid price. In order to avoid the potential enumerator's intentional bias in selecting the second bids, we assigned a random number to each level of second bids in electronic devices such that the enumerators were unaware of the second bid until they selected the random number.

Based on the framework described above and the bids, the range of WTP values were estimated for “yes” responses in the range of (P,+∞), for “no” – “yes” responses in the range of $\left(P_{d},P\right)$, and for “no” – “no” responses in the range of $\left(0,P_{d}\right)$. Where, P is the initial bid price and $P_{d}$ is the follow-up bids. In our case, for the farmers who responded against the first and the second bids, we assumed their minimum WTP as “zero.” Hence, the three probabilities for bid responses can be represented as:

(1) P(Yes)=Prob(WTP≥P)

(2) $$P\left(No-Yes\right)=\text{Prob }\left(WTP\leq P\right)-\left(\text{WTP}\leq P_{d}\right)$$

(3) $$P\left(No-No\right)=\text{Prob}\left(WTP\leq P_{d}\right)$$

The likelihood functions from the range of above-described values for the WTP model can be estimated in the following way:

(4) $$\text{ln }L=\overset{\text{N}}{\underset{\text{i}=1}{\sum }}d^{Y}\text{ln}\left[1-\text{Φ}\left(\frac{P-\beta^{'}x}{\varepsilon }\right)\right]+d^{NY}\text{ ln}\left[1-\text{Φ}\left(\frac{P-\beta^{'}x}{\varepsilon }\right)-\text{Φ}\left(\frac{P_{d}-\beta^{'}x}{\varepsilon }\right)\right]+d^{NN}\text{ ln}\left[\text{Φ}\left(\frac{P_{d}-\beta^{'}x}{\varepsilon }\right)\right]$$

Here, $d^{Y}$, $d^{NY},$ and $d^{NN}$ are binary indicator variables for three response groups and x is the vector of household-level socioeconomic attributes that are likely to influence WTP for the mini-tiller technology. The parameter ε is the standard error of the regression, which captures the randomness in the bid function. The estimation coefficient β can directly interpreted as the marginal effect of the variable x on WTP. The mean WTP is obtained by evaluating the estimated coefficient at variables mean values.

## 4 Results and discussion

### 4.1 Farm characteristics

The socio-economic attributes of farm households play an important role in influencing the WTP for the technology. Farmers' household socio-economic attributes differ across different socio-economic strata. We present the variation in household-level attributes across the lowest and top quartile farms with variable farm size. Our results show that only 28.7% of the farmers responded positively to the initial price (Table A1). Among the farmers who responded positively to the initial bid price, almost 29% were from the top quartile farms, while only 13% were from the lowest quartile farms. During the successive follow-up bids, almost 43% of the farmers responded positively to the bids, indicating the demand for the mini-tiller is price-sensitive.

Summary statistics of farm characteristics segregated by landholding classes are presented in Table 1. The average land holding of the surveyed households is only 0.4 ha. The bottom quartile farms have 0.12 ha of land while the top quartile farms have significantly more land at 0.85 ha; hence, we expect that farm size would influence the demand for farm mechanization. On average, sampled household heads were 48 years old, with only five years of formal schooling. Nearly 48% of the surveyed farmers belonged to the general caste category, and the general caste was more prevalent in the top quartile farms.^4^ Almost four-fifths of households were headed by males, and the percentage of male-headed households was lower among the bottom quartile farm households compared to the upper quartile. The primary occupation of almost 58% of household heads was farming, and a significantly higher percentage of top quartile farms were involved in on-farm activities. On average, farmers had 26 years of farming experience, and top quartile farmers were more experienced than bottom quartile farmers. While the average household size overall was 5.6 members, top quartile farms had a significantly higher number of household members than the bottom quartile farms. Due to the migration of a large number of family members in the bottom quartile farms, no significant difference in off-farm income across the top and bottom quartile farms was detected. These results indicate that farmers belonging to the bottom quartile mostly rely on off-farm activities for income generation.

**Table 1:** Descriptive statistics of the variables used to assess willingness to pay.

Variables	Overall sample (N = 628)		Bottom quartile farms (N = 157)		Top quartile farms (N = 192)		Sig.
	Mean	Std. Dev	Mean	Std. Dev	Mean	Std.	
						Dev	
*Land and livestock*							
Farm size (ha)	0.41	0.46	0.12	0.04	0.85	0.63	***
No of bullocks holding (no)	0.53	0.91	0.36	0.78	0.79	1.05	***
*Demographic*							
Age of household head (years)	48.22	11.22	46.89	11.95	50.70	11.28	***
Sex of household head (1 = male, 0 = otherwise)	0.79		0.69		0.87		***
Household size (no)	5.56	1.96	5.13	1.70	5.90	2.09	***
Caste of household (1 = general caste, 0 = otherwise)	0.42		0.36		0.51		***
*Human capital*							
Education of household head (years)	5.00	4.24	4.41	3.88	4.48	4.72	
Occupation of household head (1 = farming, 0 = otherwise)	0.58		0.52		0.67		***
Years of farming (years)	25.74	12.03	24.83	12.81	28.39	12.85	***
Household members migrated (no)	0.38	0.59	0.46	0.65	0.33	0.54	**
*Income and wealth*							
Off-farm income (‘000 *NPR*)	293.57	229.81	313.13	199.57	314.50	295.63	
Own pumps (1 = yes, 0 = otherwise)	0.22		0.12		0.28		***
On-farm labor wage rate (NRs)	630.10	201.61	564.32	176.84	658.92	210.81	***
*Access to facilities and membership*							
Market distance (kilometers)	11.04	8.92	12.25	8.95	8.29	7.46	***
Credit access (1 = yes, 0 = otherwise)	0.94		0.90		0.93		
Mobile phone holding (1 = yes, 0 = no)	0.94		0.92		0.95		
Groups/Cooperatives membership (1 = yes, 0 = no)	0.63		0.53		0.66		***
Household food security status (1 = food deficit, 0 = food secured)	0.73		0.93		0.45		***
Difficulty finding laborers (1 = yes, 0 = otherwise)	0.12		0.11		0.13		
Availability of bullocks (1 = difficult, 0 = otherwise)	0.15		0.16		0.12		
*Location*							
EH (1 = household located in eastern hills, 0 = otherwise)	0.10		0.01		0.25		***
FWH (1 = household located in far-west hills, 0 = otherwise)	0.01		0.00		0.02		*
WH (1 = household located in western hills, 0 = otherwise)	0.17		0.21		0.17		
MWH(1 = household located in mid-west hills, 0 = otherwise)	0.08		0.05		0.15		***
CH (1 = household located in central hills, 0 = otherwise)	0.64		0.73		0.42		***
*Farmer by types of crop grown*							
Rice grower (1 = yes, 0 = no)	0.51		0.38		0.63		***
Maize grower (1 = yes, 0 = no)	0.74		0.80		0.63		***
Wheat grower (1 = yes, 0 = no)	0.32		0.23		0.31		
Vegetable grower (1 = yes, 0 = no)	0.32		0.29		0.39		

*, **, and *** denote significantly different across the bottom and top quartile farms at 10%, 5%, and 1% levels, respectively. Exchange rate 1 US $ = NPR 104 during the survey year [65].

Market infrastructure is less developed in hilly areas of Nepal. Thus, the average distance to input markets in the surveyed area is about 11 km, and households in the top farm quartile were located closer to the input markets. Almost 63% of farm households are associated with farmer groups or cooperatives, and over 94% of farm households have access to credit services and own mobile phones. Farmer groups and cooperatives in Nepal are mostly centered on saving and credit services, which could be one of the reasons for the greater access to credit services. Ownership of household farm assets such as irrigation pumps is also higher for the top quartile farms, although only one-fifth of total sampled farms own pumps. Households in the mid-hills of Nepal are subsistence farmers and produce agricultural outputs primarily for their own household consumption. Our results show that almost 73% of farm households said that the crop produced is not sufficient for their household, making them food insecure. Food insecurity is more pronounced for the farms in the bottom quartile.

Labor out-migration has resulted in a shortage of agricultural labor, causing increases in labor wages. Almost 12% of respondents replied that they faced difficulty finding laborers. On average, wage rates are NPR 630 (US $6.06) per day for a laborer, and the top quartile farms were paying significantly higher labor wages than the bottom quartile farms. Land preparation for agricultural crops in Nepal is generally achieved using bullocks, and 15% of farmers reported difficulty in renting bullocks.

Farmers in the mid-hills of Nepal grow different crops in different seasons, and crop rotations and types of crops grown by the farmers may influence WTP for mini-tiller technology. Almost three-fourths of farmers grow maize, and almost half of the farmers grow rice. Maize is a major crop for the majority of the farmers in the bottom quartile farms while rice is the major crop for the majority of farmers in the top quartile. About one-third of the farms grow wheat and vegetables. A higher percentage of top quartile farms grow wheat and vegetables than the bottom quartile farms. The spatial variation in farm size quartiles is also detected across the developmental regions in the hills. Farm households in the western and central hills mostly fall in the bottom quartile farms.

### 4.2 Factors influencing a farmer's willingness to pay for the mini-tiller

We specified two WTP models. Model-I consists only of the farm-level socio-economic attributes, while Model-II includes farmer cropping systems. The results from the two models are quite consistent regarding the coefficients signs and significant levels. The model results from interval regression as specified in equation (4) are presented in Table 2. The results show that increasing farm size plays a positive role in influencing WTP for mini-tillers. However, the coefficient of farm size squared is negative, indicating that the added farm size diminishes the WTP for mini-tillers after a certain level of farm size of 4.94 ha.^5^ Our study demonstrates that even in smallholder farming systems (with an average farm size of less than 0.5 ha), farm size plays an influencing role in determining farmers' WTP for small-scale farm mechanization. These results are similar to the earlier studies that have shown that farm size is the most important determinant for the adoption of farm mechanization technologies in developing countries [3,[50], [51], [52]].

**Table 2:** Willingness to pay interval regression estimation results.

Variables	Model-I			Model-II		
	Coefficient	Std. error	*P *>* z*	Coefficient	Std. error	*P *>* z*
*Land and livestock*						
Farm size (ha)	19,692.7	4,760.0	***	19,058.8	4,756.4	***
Farm size squared	−3,982.9	1,575.4	***	−3,867.1	1,561.1	***
Bullocks holding (no)	−3,114.4	1,067.5	***	−2,758.6	1,095.4	***
*Demographic*						
Age of household head (years)	84.3	167.4		94.0	166.3	
Sex of household head (1 = male)	4,839.1	2,256.1	**	4,558.5	2,242.6	***
Household size (no)	820.1	545.8	*	710.2	542.9	
Caste of household (1 = general caste)	2,252.6	1,855.1		928.5	1,882.7	
*Human capital*						
Education of household head (years)	72.2	264.7		−12.3	264.1	
Occupation of household head (1 = farming)	−284.0	1877.5		−728.9	1865.2	
Years of farming (years)	−8.2	152.8		1.4	151.8	
Household members migrated (no)	3,630.2	1,671.1	***	3,410.1	1,654.8	***
*Income and wealth*						
Off-farm income (*NPR*)	-7E-03	5E-03		-5E-03	5E-03	
Owning engines/pumps (1 = yes, 0 = otherwise)	5,864.3	2,253.1	***	5,649.5	2,239.7	***
Bullock availability (1 = difficult)	2,385.6	2,615.9		2,055.8	2,593.0	
On-farm labor wage rate (*NPR*)	21.8	6.5	***	21.5	6.6	***
*Access to facilities and membership*						
Market distance (kilometers)	566.5	138.6	***	593.7	138.2	***
Credit access (1 = yes)	6,725.8	3,950.8	*	5,969.6	3,935.7	*
Mobile phone owning (1 = yes)	−517.4	3,785.5		72.9	3,760.8	
Group/cooperative membership (1 = yes)	4,137.0	1,969.6	***	3,946.8	1,952.0	***
Food security status (1 = food in-secured)	3,358.6	2,236.0	*	4,423.7	2,283.1	*
Labor availability (1 = difficult)	4,135.2	2,885.3		4,233.7	2,862.6	*
*Location*						
EH (1 = eastern hills)^#^	15,261.9	3,601.8	***	18,739.5	3,791.3	***
FWH (1 = far-west hills)	7,162.7	8,346.7		6,005.8	8,347.0	
WH (1 = western hills)	10,417.3	2,776.8	***	11,120.4	2,819.3	***
MWH (1 = mid-west hills)	14,840.5	3,578.8	***	14,193.2	3,624.7	***
*Farmers by types of crop grown*						
Rice grower (1 = yes)	–	–	–	3,700.3	1,972.9	*
Maize grower (1 = yes)	–	–	–	4,338.3	2,123.4	***
Wheat grower (1 = yes)	–	–	–	2,735.8	1,975.2	
Vegetable grower (1 = yes)	–	–	–	5,072.3	19,56.1	***
Model intercept	−16,936.4	8,468.5	***	−24,656.4	8,739.0	***
Log likelihood	−575.7			−569.7		
LR- *χ*^2^ [df]	144.9 [25]		***	156.8 [29]		***
Number of observations	628			628		

*, **, and *** denote significantly different across the bottom and top quartile farms at 10%, 5%, and 1% levels, respectively. ^#^central hills is the base category. Exchange rate 1 US $ = NPR 104 during the survey year [65].

The coefficient of households' member migration status is statistically significant and positively associated with the WTP for mini-tillers. The plausible explanation for this could be labor shortage at the household level. This could also be explained by the fact that the household with migrated members receive remittances and face fewer financial constraints. This can be further verified from the significant positive WTP associated with difficulty in findings laborers, particularly during the land preparation time. Furthermore, the coefficient of wage rates also has a positive impact on the demand for mini-tillers. Our results suggest that the demand for mechanization increases in response to labor shortages and rising costs of production.

The reasons for the positive association of these variables with WTP for small-scale mechanization in these smallholder systems could be due to labor scarcity at the household level [18,19], rising rural wages [21,25], and scarcity in draft animals due to decreasing trends of keeping draft animals [28,53]. It should be noted that farmers in Nepal have started to leave cultivated land fallow due to the higher cost of production driven by increasing wages and acute labor shortages [21,22,54]. Nevertheless, the negative association of the number of draft animals with mini-tillers demand is plausible. As the draft animals and mini-tiller are mostly used for agriculture land preparation, the negative association of WTP for mini-tillers, and the number of draft animal owned is expected.

Unlike earlier studies that have demonstrated a negative association between distance to market and probability for technology adoption [[55], [56], [57], [58], [59]], our results show that the WTP for mini-tillers increases with an increase in market distance.^6^ It is plausible to assume that the market infrastructure in hilly areas of Nepal is less developed and local markets in rural areas charge a higher price for any novel technology than the prevailing technology prices in the large and urban-centered markets; hence, most of the new technology in Nepal is distributed from urban-centered markets to the rural markets. Mini-tillers are mostly imported from China by Nepalese importers from large city areas and then distributed to the local traders in the local village markets after adding profit margins and transportation costs.

Other factors that positively influenced the WTP for mini-tillers are group and cooperative membership, credit access, male-headship of the households, household food-insecurity status, and irrigation pump ownership. Demand for the mini-tiller is higher among male-headed households, as it is difficult for females to use mini-tillers due to socio-cultural reasons.^7^ The access to credit positively influenced the farmer's WTP for the mini-tiller, as access to credit eases the financial constraints that rural households normally face. The result also indicates that farmers with greater credit access are linked with groups and cooperatives and have a positive association with WTP for mini-tillers. In Nepal, association with groups and cooperatives and access to credit services is interlinked. Most of the sampled groups and cooperatives conduct saving, and credit activities for their members, and these groups/cooperatives are linked with the formal banking systems either to deposit savings or credit lending [60]. Our results are supported by the earlier studies that have demonstrated the positive relationship between technology adoption and access to credit services [[61], [62], [63], [64]]. Similarly, household ownership of the irrigation pump also positively influences the WTP for the mini-tiller, as it clearly reflects the household ability to pay for technology.

The WTP for mini-tillers is also positively influenced by the types of crop grown by the farmers. Results show that farmers growing rice, maize, and vegetables have a higher WTP for mini-tillers since these are labor-intensive crops. Furthermore, the spatial heterogeneity in WTP for mini-tillers is evident with higher demand in the eastern, western, and mid-western hills.

### 4.3 Demand heterogeneity

The average WTP for mini-tillers across farm size quartiles and developmental regions are presented in Table 3, and results are based on a prediction from the WTP interval regression model as specified in Table 2 (Model-II). Results show that the average WTP for mini-tillers was NPR 38,193 (US $367) and this amount was 31% lower than the actual market price of the mini-tiller. Only 6% of farmers expressed their WTP for the mini-tiller technology at the average market price (NPR 55,000 or US $529), and all these farms fall in the top quartile of farm size. Furthermore, the farms in the bottom quartile were willing to pay 26% less for the mini-tillers than top quartile farms. The top quartile farms from all the developmental regions were willing to pay a higher price for the mini-tillers than the farms in the bottom quartile. Our results confirm that the demand for the farm mechanization by the smallholder is higher when the price of the technology is lower than the current market price.

**Table 3:** Estimated WTP (*NPR*) for mini-tillers across different farm size quartiles and hilly regions.

	Overall farms		Bottom quartile farms		Top quartile farms	
	Mean	Std. error	Mean	Std. error	Mean	Std. error
Eastern hills (EH)	47,575	1,120	34,472	1,889	50,555***	1,193
Central hills (CH)	35,691	535	31,131	863	38,588***	1,096
Western hills (WH)	38,595	868	35,928	1,110	43,394***	1,672
Mid-west hills (MWH)	45,464	1,257	37,029	1,870	47,707***	1,622
Far-west hills (FWH)	35,450	2,207	–	–	39,582	3,134
Overall mid-hills	38,193	435	32,483	697	43,712***	751

*** indicate significantly higher than the smallest 25% of farms at 1% level of probability. Exchange rate 1 US $ = NPR 104 during the survey year [65].

**Fig. 2:**
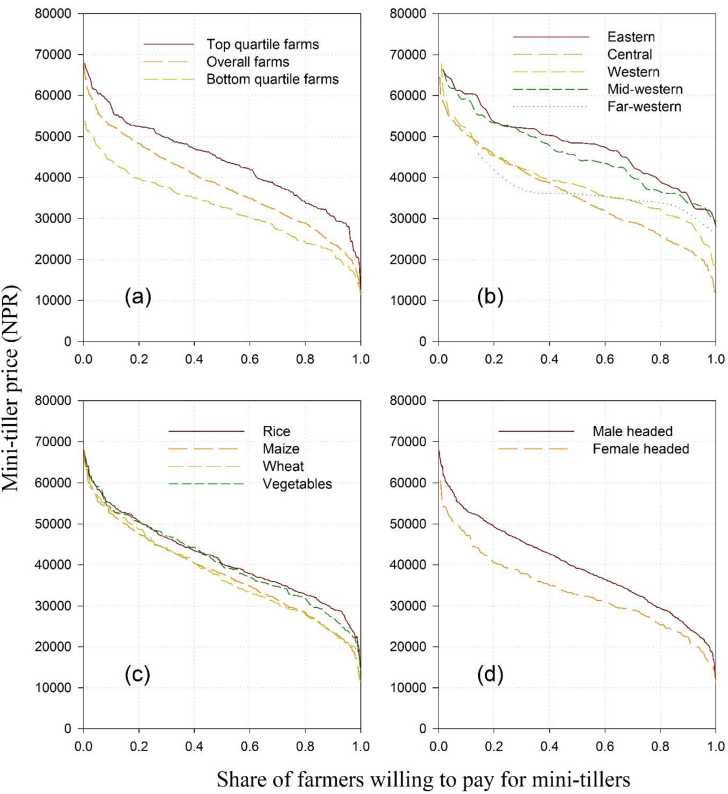
Demand curves for mini-tillers across; (a) farm size quartiles, (b) hilly regions, (c) crop types, and (d) household gender.

The demand curves for mini-tiller technology across different socioeconomic strata are presented in Fig. 2. Overall, all the demand curves were elastic, and the demand for mini-tillers for the farmers located in the far-western development regions was highly elastic compared to other regions, possibly due to high poverty rates in these regions. Farmers in the eastern and mid-western hills had a higher demand for the mini-tillers than the farmers in other regions, although the average WTP was lower than the average market price in all the regions. Furthermore, farms growing rice and vegetables had a higher demand for the mini-tiller technology than the farmers growing wheat and maize, which in general requires fewer laborers. Finally, households with a male as the primary decision maker had a higher demand for the mini-tiller technology than female-headed households. Our results indicate that decreasing the technology price could help the spread of scale-appropriate farm mechanization, or the development of service provision models [23] where individual mini-tiller owners provide services to other farmers could increase technology adoption levels and rural entrepreneurship development.

Results from our study suggest that government cost-sharing programs should be targeted at the bottom quartile farms, thus making them the mini-tiller service takers and service providers. However, increasing the mini-tillers market access should be the priority for the farms with high WTP. These policies would promote an increase the level of farm mechanization in the country. Moreover, the results on differential demand curves could be useful for the private sector in quantifying the potential market for mini-tillers in different developmental regions.

## 5 Conclusion and policy implications

Agricultural technologies have a high potential to mitigate the challenges posed to farming communities in developing countries, and the adoption of such technologies increases the welfare of the societies. Farm mechanization based technologies play a crucial role in attenuating the problems associated with farm drudgery, high cost of cultivation, low productivity, and labor scarcity. In the recent years, farming communities in developing countries have been facing acute labor shortages due to out-migration, which has affected farm productivity and profitability. Finding scale-appropriate farm mechanization based options to cope with such problems is a major priority for policymakers. As such, labor scarcity in Nepal has affected smallholder farming systems by increasing labor prices, thereby negatively affecting farm enterprise returns, profits, and productivity. In this context, the current study assessed smallholder farmers' WTP for scale-appropriate farm mechanization by taking the case of mini-tiller technology in the mid-hills of Nepal. This study would be the first to assess the WTP for scale-appropriate farm mechanization in developing countries with a high rate of labor out-migration.

Our results show that farm size, on-farm wage rates, number of household members migrated, access to credit services, and association with cooperatives were the farm-level attributes that are positively associated with WTP for mini-tillers. However, farms holding a higher number of draft animals are negatively associated with WTP for the purchase of mini-tiller technology. Other household attributes that influenced WTP for mini-tillers are pumsets ownership, having a male as the household head, distance to markets, and the types of crops grown such as maize and vegetables. Moreover, our results demonstrate that farmers' average WTP was 31% lower than the actual price of a mini-tiller. Findings from this study show a heterogeneous demand for mini-tillers across different socioeconomic strata. The smallest quartile farms were willing to pay a 26% lower price for the technology compared to top quartile farms.

Results from this study suggest that the government of Nepal can formulate different farm mechanization sectorial development strategies by devising different programs based on the demand for mini-tillers across different socioeconomic strata. Since our study has shown a substantially lower WTP for the mini-tillers at the current market price, we suggest the development of a service provision model in which multiple farms can benefit through hiring mini-tiller services. This would increase the likelihood of technology adoption. Finally, government policies in Nepal should aim to develop targeted cost-sharing programs and service provision models to increase the level of mechanization in the country.

## Conflicts of interest

We declare to have no conflict of interest.

## Figures and Tables

**Table A1:** Bid structure for willingness to pay elicitation.

	Discount (%)	Bid (NPR)	Yes-response		No-response	
			No. of households	%	No. of households	%
Initial bid (overall)	0	55,000	155	24.68	473	75.32
Initial bid (bottom quartile farms)	0	55,000	21	13.38	136	86.62
Initial bid (top quartile farms)	0	55,000	55	28.65	137	71.35
Second bid^a^						
Bid 1	9.09	50,000	1	24.84	24	75.16
Bid 2	12.73	48,000	1	25.00	19	75.00
Bid 3	16.36	46,000	3	25.48	30	74.52
Bid 4	20.00	44,000	11	27.07	36	72.93
Bid 5	23.64	42,000	7	28.18	50	71.82
Bid 6	27.27	40,000	12	30.10	40	69.90
Bid 7	30.91	38,000	4	30.73	34	69.27
Bid 8	34.55	36,000	5	31.53	9	68.47
Bid 9	38.18	34,000	10	33.12	9	66.88
Bid 10	41.82	32,000	13	35.19	19	64.81
Bid 11	45.45	30,000	27	39.49	48	60.51
Bid 12	49.09	28,000	20	42.68	41	57.32

a The responses were elicited from farmers who answered negatively against the initial bid. Exchange rate 1 US $ = NPR 104 during the survey year [65].

## Footnotes

1 Scale-appropriate farm mechanization is defined as the mechanization options that best fit the social, economic, and production ecology characteristics of different farm types.

2 Nepal has five developmental regions (eastern, central, western, mid-western, and far-western). The selected districts were Doti, Surkhet, Palpa, Nuwakot, Kavre, and Illam. We also selected two districts from the central development region due to a higher potential for mini-tiller adoption based on the cultivated area.

3 Wards are the smallest administrative units and we have considered wards as the villages for the purpose of this study. The number of wards in each sub-district ranges from 9 to 20.The number of villages selected ranges from one to nine, depending on the potential for mechanization.

4 In Nepal different socially determined caste systems exists. Broadly, there are four types of caste, Brahmin, Chettri, Vaish and Shudra. The Brahmin are consider as the upper caste, while the Sudra are considered as untouchable and lower caste. In this study we have considered Chettri and Vaish as the general caste. For more detail see Mosse [66].

5 Condition for maximum fulfilled for farm size = 19,692.7/3,982.9 = 4.944 ha (the coefficients are shown in Table 2).

6 One of the reviewers suggested that the distance to market may also affect the WTP for the mini-tiller services. However, our study assumed the availability of the mini-tillers in the local-markets and assessing farmers WTP for the mini-tiller services is beyond the scope of this study.

7 In Nepalese society, women are banned from using the plough. If females use the traditional plough for land preparations or tillage, it is highly criticized and blamed for low productivity.
